# Effects and meanings of a person-centred and health-promoting intervention in home care services - a study protocol of a non-randomised controlled trial

**DOI:** 10.1186/s12877-017-0445-0

**Published:** 2017-02-16

**Authors:** Karin Bölenius, Kristina Lämås, Per-Olof Sandman, David Edvardsson

**Affiliations:** 1 Department of Nursing, Umeå University, 90187 Umeå, Sweden; 2 School of Nursing and Midwifery, La Trobe University, Melbourne, Australia; 3 Division of Caring Sciences, Department of Neurobiology, Care Sciences and Society, Karolinska Institutet, Stockholm, Sweden

**Keywords:** Aged care, Decision making, Health, Home care services, Older people, Person-centred, Quality of life

## Abstract

**Background:**

The literature indicates that current home care service are largely task oriented with limited focus on the involvement of the older people themselves, and studies show that lack of involvement might reduce older people’s quality of life. Person-centred care has been shown to improve the satisfaction with care and quality of life in older people cared for in hospitals and nursing homes, with limited published evidence about the effects and meanings of person-centred interventions in home care services for older people. This study protocol outlines a study aiming to evaluate such effects and meanings of a person-centred and health-promoting intervention in home aged care services.

**Methods/design:**

The study will take the form of a non-randomised controlled trial with a before/after approach. It will include 270 older people >65 years receiving home care services, 270 relatives and 65 staff, as well as a matched control group of equal size. All participants will be recruited from a municipality in northern Sweden. The intervention is based on the theoretical concepts of person-centredness and health-promotion, and builds on the four pedagogical phases of: theory apprehension, experimental learning, operationalization, and clinical supervision. Outcome assessments will focus on: a) health and quality of life (primary outcomes), thriving and satisfaction with care for older people; b) caregiver strain, informal caregiving engagement and relatives’ satisfaction with care: c) job satisfaction and stress of conscience among care staff (secondary outcomes). Evaluation will be conducted by means of self-reported questionnaires and qualitative research interviews.

**Discussion:**

Person-centred home care services have the potential to improve the recurrently reported sub-standard experiences of home care services, and the results can point the way to establishing a more person-centred and health-promoting model for home care services for older people.

**Trial registration:**

NCT02846246.

## Background

This study focuses on older people receiving home care services (HCS) and aims to evaluate the effects and meanings of a person-centred and health-promoting HCS intervention. The intervention is person-centred in that it will focus on the older people’s expressed needs and shared decision making concerning care planning and delivery [1, 2], and it is health-promoting as it aims to enable older people to increase their control over their own health [3]. The intervention will enable the older person and family, together with a contact nurse; to have conversations about and prioritise care content that can satisfy psychosocial, physical, and functional needs of the older person.

In the literature, HCSs nationally and internationally have been criticised for not meeting the psychosocial needs of older people [4, 5]. Older people receiving care in their own homes have reported experiences of being lonely [6], isolated [4, 7], inactive, under-stimulated [4, 5] and experiences of life stress [5]. The essence of this criticism relates to perceptions that HCS are primarily granted and delivered based on physical and functional needs, while psychosocial needs and quality of life needs (QoL) have been given less priority [6]. This means that there seems to be a current gap between needs and services, between expectations and experiences, which may lead to expressions of dissatisfaction.

Satisfaction with HCS has been studied worldwide. For example, Kadowaki et al. [4] showed in a Canadian study that older people receiving HCS, who reported that their psychosocial needs were met, also reported higher levels of satisfaction compared to those with unmet psychosocial needs. Other studies report that older people have limited satisfaction with the HCS they receive [5, 7–12] with respect to: limited help in participating in social and daytime activities [5, 7, 11, 13]; detailed time-saving work schedules resulting in lack of time and stressful visits [8, 9, 11, 12]. Thus there seems to be international consensus indicating that older people experience shortcomings regarding the psychosocial aspects of the care provided by HCS.

In addition, shared decision making and influencing the planning of HCS have been reported as important for older people, for example from studies in Australia and Europe [14–19]. Hautsalo et al. [15], Gill et al. [14] and Kaambwa et al. [17] showed that older people wished for a flexible and adaptable HCS that meets their individual needs. It was described as important to choose and influence daily activities. A trusting relationship between the older person and professional staff is described as essential to enable shared decision making to facilitate the older person’s autonomy and sense of being respected [16, 18, 19]. However, both international [16, 20] and Swedish studies [8, 21, 22] report limited possibilities for older people to make shared decisions about their content of care in HCS. Such findings indicate a need to learn more about the effects and meanings of providing HCS that allows shared decision-making.

Furthermore, it has also been found that quality of care is related to QoL among older people receiving HCS [23]. Low QoL has been found to be associated with reduced ADL function, depression, loneliness, not being engaged in meaningful activities [6, 24, 25], social isolation [24, 26] and pain [6]. However, it has also been shown that high quality care can compensate for the deficiencies mentioned above and result in an improved QoL [23, 27]. It could be hypothesised that when older people with HCS have the opportunity to influence the content of care through shared decision-making, the chances of them having their needs met increase, as does their QoL.

In addition, relatives have an important role in the HCS [28–30] and is often there to assist the older person for example with medications, household duties and personal care [29]. Reports from relatives indicate that they often become more engaged with the older person than what they can manage [30], indicating a strong caregiver burden. In addition, the contact with formal care is often initiated by relatives, who often describe themselves as being the bridge between professional staff and the older person, suggesting that care should be a shared responsibility between the older person, relatives and care staff [30]. Such findings indicate the importance of including the family in interventions, and that implementing shared decision-making might improve relatives’ satisfaction with care as well as reducing caregiver strain.

When it comes to age care staff in home care services, a growing crisis has been described with high turnover rates and challenges in recruiting and retaining skilled care professionals. Staff have reported experiences of dissatisfaction and frustration with work [31–33] with respect to a stressful and hectic work situation [31, 33], increasing demands for efficiency [31], not having sufficient time to converse, provide the “little extras”, and provide care in the psychosocial domain [32]. Not being able to provide care in a way that is perceived as satisfactory has been shown to be a predictor of stress of conscience among care staff [34], and a Swedish study showed that the prevalence of stress of conscience has increased among aged care staff in recent years [35].

A more person-centred approach that builds on shared decision making with care recipients and that systematically explores and documents the person’s subjective experiences, expectations, preferences and needs, and incorporate these in care plans and delivery has been shown facilitating high quality care [36, 37]. There is evidence to suggest that older people in person-centred nursing homes had higher QoL compared to older people in less person-centred nursing homes [38], and older people with mild dementia living at home and receiving PCC and collaborative day-care program described having more meaningful lives and increased wellbeing [27]. Other studies in aged care have shown positive results from implementing a person-centred approach [39–41]. For example, a study that implemented PCC in rehabilitation care resulted in functional improvements and higher satisfaction with care [41]. In addition, Olsson et al. [39] showed that increased involvement in care resulted in lower costs, higher physical function, and shorter stays in hospital after hip fractures. Furthermore, Rokstad et al. [40] showed that PCC of older people with dementia can prevent and reduce agitation and depression. Thus, it seems reasonable to interpret that more person-centred and health-promoting home care services can have the potential to increase shared decision-making, increase the focus on psychosocial issues of care, and increase QoL, thriving and satisfaction with care. Even though few intervention studies exist that have evaluated effects from PCC models on wellbeing and experiences of staff in home care services, the evidence for positive effects such as reduced job stress and strain [42], increased personal and professional satisfaction, [43], and less emotional exhaustion [44] in nursing homes have been reported. The evidence for effects of PCC on relatives’ experiences of home care services is sparse and needs further exploration.

### Rational

The literature review above indicates that current HCS is largely oriented towards physical function and the completion of care tasks, with limited focus on psychosocial needs and on involving older people in care planning and decision making, which leads to dissatisfaction with care and low QoL. The literature also describes home care services as being demanding and challenging to both relatives and staff. This study will explore the extent to which a person-centred and health-promoting intervention focusing on psychosocial needs and shared decision- making can improve QoL and satisfaction with care for older people, as well as improve the experiences of relatives and staff.

### Overall aim

The study in this protocol aims to evaluate the effects and meaning of a person-centred and health-promoting HCS intervention on QoL, thriving and satisfaction with care in older people, on caregiver strain, informal caregiving engagement and satisfaction with care among relatives, and on job satisfaction and stress of conscience among care staff.

### Research questions

1. To what extent will the intervention have significant effects on
  QoL, thriving, and satisfaction with HCSs in older people?
  Caregiver strain, informal caregiving engagement and satisfaction with care among relatives?
  Job satisfaction and stress of conscience among care staff?
2. What are the experiences and meanings of the intervention as narrated by older people, relatives and care staff?

## Methods/design

A non-randomized controlled trial with a before/after design will be used to explore the effects of the intervention, and a phenomenological-hermeneutic approach to illuminate meanings of the intervention.

### Participants and setting

All participants will be recruited from one municipality in northern Sweden. Two hundred and seventy older people from one geographical HCS district will be invited to form the intervention group and 270 older people from a different geographical HCS district will be invited to form the control group. Both districts have similar organisations, working conditions, staff skill mix and educational levels. In addition, one relative for each person receiving HCS (*n* = 540) will be asked to participate. Sixty-five HCS staff will be invited to participate in the intervention group and 65 in the control group. The inclusion criteria for the older person will be: 1) aged 65 years or older; 2) living at home and receiving HCS, with at least two visits per month and 3) able to speak Swedish. Inclusion criteria for relatives will be; 1) defined by the older person as his/her relative; 2) able to speak Swedish. Inclusion criteria for staff will be; 1) employed in the HCS district for more than 6 months at baseline, as contact staff and 2) able to speak Swedish. A convenient sub-sample of older people (*n* = 30), relatives (*n* = 30), and HCS staff (*n* = 30) from the intervention group will be invited to participate in qualitative research interviews.

### Power calculations

Sample size calculations for the primary endpoint (NHP) [45], for older people indicate that a sample of *N* = 207 will provide 85% power at the 0.05 significance level to detect case control mean differences of 6, based on previously reported results on the dimension social isolation [46]. Further power calculations indicate that the study is sufficiently powered to detect significant differences for the other National Health Profile (NHP) dimensions and also for the secondary endpoints. To adjust for a drop-out rate of 29% based on study by Rokstad et al. [40], 270 participants will be recruited.

### Intervention

The intervention is based on the theoretical concepts of person-centredness [1, 2] and health-promotion [3] and builds on four pedagogical phases; theory apprehension, experimental learning, operationalization, and clinical supervision. The content of these phases is further described below. The first intervention phase (10 months) will be followed by a second implementation phase in which the HCS will take over the continuation of the intervention with the research team providing support.

#### Theory apprehension

Firstly, staff will engage in a 90-min web-based educational program on the content, meaning, operationalization and outcomes of the central theoretical components of person-centredness [1, 2], person-centred health and care conversations [47] and the evidence-based and nursing knowledge underpinning these concepts. The purpose is to increase knowledge about these concepts, apply theoretical knowledge to daily work, and gain the skills needed later to complete the person-centred health and care conversations with the older person and their relatives. The web-based part includes video lecturers and self-reflective activities.

#### Experimental learning

Secondly, staff will participate in a seminar (180 min) including supervised skills training, based on Kolb’s experimental learning model [48], in how to explore the needs and wishes of the person receiving care with person-centred health and care conversations. Kolb describes learning as a circular movement between experience and reflection. To develop their skills the staff will engage in role-play interspersed with reflective questions such as: What happened in the conversation? What does it mean? What can I learn from that? How can I use what I have learned?

#### Operationalization

Thirdly, staff will conduct a person-centred health and care conversation (60 min) with each older person participating in the study. The purpose of these conversations is to evaluate the extent to which current HCS practices meet the older person’s needs, to maintain or rearrange the care plan to provide care that maximises older people’s health and satisfies psychosocial as well as physical needs. The conversation will be documented and used to influence changes in the care plans as well as in daily care. In addition, staff will be encouraged to balance the care plans with the daily needs and priorities of the older person, so that the daily work of staff will be increasingly characterised by flexibility in adapting planned activities to the older person’s current needs. The person centeredness will thus influence both the planning and daily provision of care. For example, showering could be replaced by a visit to the library if the older person regards the latter as being significantly more conducive to his/her QoL at the moment.

#### Clinical supervision

Finally, staff will participate in ten group supervisory sessions over the course of seven months. The aim is to support and facilitate ongoing operationalization of the central concepts of person-centred and health-promoting care and to influence practice change for each person receiving HCS by discussing facilitators and barriers to such changes and how to reflect on or resolve these in clinical practice.

### Control group

The control group will be offered one 30-min on-line lecture with state-of-the-art knowledge on contemporary care for people with dementia. A ‘usual care paradigm’ will guide the control units, i.e., a continuation of practice as usual. Control units will receive the intervention protocol and study results at the end of the study.

### Data collection and procedures

Data will be collected by means of self-reported study questionnaires and qualitative research interviews.

#### Questionnaires

Older people receiving HCS, their relatives and care staff (intervention and control groups) will be asked to complete a questionnaire covering demographics and study endpoint variables at baseline and at 12- and 24-month follow-up. Study outcomes for older people, relatives and staff will be measured using the following questionnaires, see Table 1.

**Table 1:** Overview of participants, outcomes and questionnaires

Participants	Outcome	Questionnaires
Older people	Health and QoL (primary outcomes)	Nottingham Health profile, EQ-5D
	Thriving	The Thriving of Older People Assessment Scale
	Satisfaction with care Involvement in decisions and care Personal activity of daily living	Quality of Care from the Patient’s Perspective Impact on Participation and Autonomy – Older Persons The KATZ ADL index
Relatives	Caregiver burden	Caregiver Burden Scale
	Satisfaction with care	The Pyramid Questionnaire
	Informal caregiving engagement	The Resource Utilization in Dementia
Personnel	Job satisfaction	The Measure of Job Satisfaction
	Stress of conscience PCC	The Stress of Conscience Questionnaire The Person-Centered Care Assessment Tool

#### Primary outcomes

Two primary outcomes will be measured, self reported health and QoL. The first primary outcome will be measured using the Nottingham Health Profile, which is a scale that includes 38 items and consists of 6 dimensions: energy level; pain; emotional reaction; sleep; social isolation; and physical abilities. Each item is presented as a statement with a Yes/No response and the score ranges from best (0) to worst (100) possible [45]. The Nottingham Health Profile has been found to be sensitive to change, and both valid [45] and reliable [49].

The second primary outcome will be measured using the EQ-5D scale, which comprises two parts, a state of health description, which includes 5 items and a visual analogue scale. The state of health description comprises five dimensions: mobility; self-care; usual activities; pain/discomfort; and anxiety/depression. Each dimension is scored on a five-level Likert-scale ranging from none (0) to extreme (4). The visual analogue scale rates participants’ overall health between endpoints, from worst imaginable health (0) to best imaginable health (100). EQ-5D has been found to be both sensitive to change and valid [50].

#### Secondary outcomes

Ten secondary outcomes will be explored, thriving, quality of care, impact on participation and autonomy, ADL-function, caregiver burden, satisfaction, resource utilisation, job satisfaction, stress of conscience and person-centredness of care. The first secondary outcome will be measured using the Thriving of Older People Assessment Scale which includes 32 items and consists of five sub-scales: resident attitude towards the place they were currently living in; quality of the care and care-givers; activities and peer relationships; opportunities to keep in touch with people and places of importance; and qualities in the physical environment. Each item has six possible responses on a Likert-scale, ranging from No (1) to Yes, I agree completely (6). The Thriving of Older People Assessment Scale has been found to be valid and reliable [51].

The second secondary outcome will be measured using the Quality of Care from the Patient’s Perspective, a scale which includes 64 items and comprises four dimensions: medical-technical competence (11 items); physical-technical conditions (10 items); identity-oriented approach (30 items); and social-cultural atmosphere (13 items). Each item is to be answered by the respondents in two ways - perceived reality and subjective importance. Perceived reality ranges from Not applicable (1) to Fully agree (5) on a five-level Likert scale while the subjective importance ranges from Of very great importance (1) to Of little importance (4). The Quality of Care from the Patient’s Perspective has been found to be valid and reliable [52].

The third secondary outcome will be measured using the Impact on Participation and Autonomy – Older Person’s questionnaire which includes 22 items and consists of eight dimensions: mobility (5 items); self-care (5 items); activities in and around the house (4 items); financial situation (1 item); financial situation (1 item); social relationship (5 items); help and support others (1 item) and summary (1 item). Each item is scored on a five-point Likert scale: very good (1) to very poor (5). The Impact on Participation and Autonomy –Older Persons has been found to be valid and reliable [53].

The fourth secondary outcome will be measured using the KATZ ADL index [54] which includes 15 items and consists of two dimensions - instrumental ADL and personal ADL. Each item is scored dichotomously dependent (0) or independent (1). The Katz ADL index has been used since 1961, and has proved consistent in evaluating functional status among older people. No formal validation studies have been found in the literature but the KATZ ADL index has been found to be reliable [55].

The fifth secondary outcome will be measured using the Caregiver Burden Scale that includes 22 items and consists of five dimensions: general strain; isolation; disappointment; emotional involvement; and environments. Response alternatives are scored on a four-point Likert-scale, ranging from Not at all (1) to Often (4). The Caregiver Burden Scale has been found to be valid and reliable [56].

The sixth secondary outcome will be measured using the Pyramid questionnaire, which includes 40 items and consists of seven parts: information; staff professional skills; care; activity; contact; social support; and relative participation. Response alternatives are scored on a four-point Likert-scale ranging from Yes, to a great degree to No, not at all. The scale has been found to be both valid and reliable [57].

The Resource Utilization in Dementia instrument will be used to measure the seventh secondary outcome and this scale includes three parts: personal activities of daily living (dressing/undressing, showering/bathing, washing, and moving); instrumental activities of daily living (cooking, shopping, washing, cleaning, taking care of finances, giving medication and transportation); and watching over (risks such as fire, fall indoors etc.). The Resource Utilization in Dementia assesses resource utilization in terms of hours of homecare, number of days in hospital, number of visits to GPs, physiotherapists, and informal care. The instrument has been found to be valid and reliable [58].

The Measure of Job Satisfaction will be used to measure the eighth secondary outcome and this scale includes 37 items and consists of five dimensions: personal satisfaction; satisfaction with workload; team spirit; training; and professional support. Responses are scored on a five-point Likert-scale, ranging from Very dissatisfied (1) to Very satisfied (5). The scale has been found to be valid and reliable [59].

The ninth secondary outcome will be measured using the Stress of Conscience scale, which consists of ten items related to various healthcare situations, each question comprises an A and a B part. The response alternatives in part A are scored on a six-point Likert-scale, ranging from Never (0) to Every day (5). The questions are related to how often different situations arise in the workplace. Part B comprises a ten-centimetre visual analogue scale where the impact of each situation on the participant’s conscience is estimated. A total index can be calculated where a higher value signifies higher levels of stress of conscience. The Stress of Conscience scale has been found to be valid [60].

Finally, the tenth and last secondary outcome will be measured using the Person-Centred Care Assessment Tool (P-CAT), a tool that consists of 13 items concerning the content of care, the environment and organization. The P-CAT will be used to evaluate changes in PCC. Response alternatives are scored on a five-point Likert-scale, ranging from Disagree completely (1) to Agree completely (5). Higher scores indicate a greater degree of PCC. The P-CAT has been found to be both valid and reliable [61].

#### Qualitative research interviews

Qualitative research interviews [62] will be utilised to explore the participants’ lived experiences of the intervention. The interviews will start with a probing statement such as: Can you please tell me about your experiences of this intervention? What has it meant to you personally? The interviews will be tape-recorded and transcribed verbatim [62, 63].

### Data analyses

#### Statistical analyses

The SPSS 22.0 for windows (SPSS Inc., Chicago, IL) will be used for all statistical analyses Data concerning the participants’ personal characteristics will be analysed using descriptive statistics. Differences between groups will be analysed using Independent sample *t*-test or the Mann-Whitney *U*-test for continuous data and the Chi-square test for dichotomised data. Changes within the two groups will be analysed separately using the paired *t*-test or Wilcoxon Signed Rank Test for continuous data and the McNemar test for dichotomised data. In addition, statistical regression models adjusting for background characteristics and account for correlated measurements for the same individual will also be used (e.g., mixed effects model and generalized estimating equations). A *p*-value of *<*0.05 will be regarded as statistically significant and effect sizes will be estimated with Cohen’s d [63]. All data analysis will be performed on an intention-to-treat principle.

#### Phenomenological-hermeneutic analysis

The transcribed interviews will be analysed using a phenomenological-hermeneutic analysis as outlined by Lindseth and Norberg [64]. Meaning units will be condensed and grouped into subthemes and thereafter abstracted into themes. Repeated structural analyses will be performed to invalidate or validate the naïve understanding, and to create a meaning structure based on the text content. The naïve understanding, structural analyses and thematic construction will then be interpreted in light of relevant literature to develop a more informed interpretation and comprehensive understanding of the meanings of a person-centred and health-promoting home care intervention.

## Discussion

The study outlined in this protocol is the first to our knowledge that aims to evaluate effects and meanings of a person-centred and health-promoting intervention in home care services. This study will target the dissatisfaction with current HCSs being task-focused and failing to meet the psychosocial and existential needs of frail older people, as well as the limited shared decision making in planning and delivery of care for older people living at home with HCS. Person-centred interventions for older people in nursing homes and home care have previously shown improvements in QoL and satisfaction with care [38–41, 65].

In previous studies [39–41, 66] among patients in hospital or the elderly living in community housing a person-centred approach has been found to have positive effects. It is thus reasonable to hypothesise that the intervention can have a similar positive effect among older people receiving HCS. The intervention will hopefully improve the health of older people and the sub-standard experiences of HCS recurrently reported, and provide further data on the development of PCC and health-promoting programs in HCS. The results will also contribute to existing international knowledge in the field as these types of studies are very limited, and may contribute to influence implementation of PCC in the HCSs.

As in other countries, aged care in Sweden is regulated by a number of laws [67, 68]. Taking the Social Service Act [67] as the point of departure, the National Board of Health and Welfare has formulated a set of national values as a basis for care delivery to older people [69]. According to these values, care should be delivered in such a way that older people can feel independent, can be supported to lead an active and meaningful life in communication with others, can live according to their own desires, and can make decisions about their care and home. In the light of recent reports highlighting a very limited shared decision making in content and planning of daily care for older people [8, 16, 20–22], a limited focus on psychosocial needs and aspects that can improve quality of life [6, 24–26], it seems utterly important to increase the knowledge regarding successful and unsuccessful interventions in this field. This study can contribute to this accumulation of evidence.

The principal limitation of this study may well be the lack of randomisation. The intervention introduces an educational programme but also a slightly new process in that person-centred health and care conversations are to be held with care recipients, and that these conversations then are to be used as facilitating change in the way care is provided in those instances that this has been regarded as needed by the participating older people and relatives. To randomize on an individual level introduces several difficulties. Firstly, it is difficult for the same care staff to adopt different procedures and switch between different care models following the randomization of individual care recipients to different groups. To switch between different care models when providing care to different people provide a risk of confusion of what care model to use and jeopardize the adherence to the scheme for intervention and control group. However, cluster randomisation would have been an option, but the municipalities that was available for participation in the study proved to be too small and therefore not able to provide the units needed to make cluster randomisation possible. It will be possible to follow up this study with a larger sample to enable cluster randomisation and higher external validity.

## Abbreviations

HCS: Home care service
PCC: Person-centred care
QoL: Quality of life
